# Why people use herbal medicine: insights from a focus-group study in Germany

**DOI:** 10.1186/s12906-018-2160-6

**Published:** 2018-03-15

**Authors:** Alexandra N. Welz, Agnes Emberger-Klein, Klaus Menrad

**Affiliations:** TUM Campus Straubing for Biotechnology and Sustainability, Weihenstephan-Triesdorf University of Applied Sciences, Petersgasse 18, 94315 Straubing, Germany

**Keywords:** Herbal medicine, CAM, Reasons for use, Focus groups

## Abstract

**Background:**

The use of herbal medicine, as one element of complementary and alternative medicine, is increasing worldwide. Little is known about the reasons for and factors associated with its use. This study derives insights for the use of herbal medicine in Germany regarding the usage aims, role played by the type of illness, reasons for preferred usage and sources of information.

**Methods:**

Using a qualitative methodological approach, six focus groups (*n* = 46) were conducted. Two groups with young, middle-aged and elderly participants, respectively. After audiotaping and verbatim transcription, the data were analysed with a qualitative content analysis.

**Results:**

We found that treating illnesses was the most frequently discussed aim for using herbal medicine over all age groups. Preventing illnesses and promoting health were less frequently mentioned overall, but were important for elderly people. Discussions on herbal medicine were associated with either mild/moderate diseases or using herbal medicine as a starting treatment before applying conventional medicine. In this context, participants emphasized the limits of herbal medicine for severe illnesses. Dissatisfaction with conventional treatment, past good experiences, positive aspects associated with herbal medicine, as well as family traditions were the most commonly-mentioned reasons why herbal medicine was preferred as treatment. Concerning information sources, independent reading and family traditions were found to be equally or even more important than consulting medicinal experts.

**Conclusions:**

Although herbal medicine is used mostly for treating mild to moderate illnesses and participants were aware of its limits, the combination of self-medication, non-expert consultation and missing risk awareness of herbal medicine is potentially harmful. This is particularly relevant for elderly users as, even though they appeared to be more aware of health-related issues, they generally use more medicine compared to younger ones. In light of our finding that dissatisfaction with conventional medicine was the most important reason for a preferred use of herbal medicine, government bodies, doctors, and pharmaceutical companies need to be aware of this problem and should aim to establish a certain level of awareness among users concerning this issue.

**Electronic supplementary material:**

The online version of this article (10.1186/s12906-018-2160-6) contains supplementary material, which is available to authorized users.

## Background

The use of complementary and alternative medicine (CAM) has continuously increased over the past decades. In their seminal paper from the late 90s, Eisenberg et al. benchmarked prevalence rates for the use of CAM-based therapy in the US [1]. They found that the use of CAM increased from 34% in 1990 to 42% in 1997 [1]. To understand this growing interest in CAM and related forms of therapies, a number of follow-up studies examined prevalence rates and use-related factors of alternative medicine [2–11]. From these studies, some common characteristics of the user of CAM can be identified. Typical users are female [2–10], middle-aged [2, 3, 5, 7, 8, 10], and well-educated [2–7, 10].

However, with respect to the ethnicity of users, their health status, reasons for use, and medical conditions for which CAM was consumed, as well as for prevalence rates within and between countries, reviews in the literature show a less consistent picture [12–14]. As cautioned by Eardley et al. [12], this could be related to an insufficiently accurate terminology, i.e. a methodology heterogeneity when including different types and numbers of therapies collectively under the ‘CAM umbrella’. Indeed, the use of different forms of alternative therapies, such as acupuncture, chiropractic or herbal medicine (HM) might be associated with different use-related factors, such as socio-demographics or health status [4, 6, 13, 15, 16] and different reasons [6, 16, 17]. Moreover, CAM prevalence rates and use-related factors are expected to vary strongly for specific countries owing to different legal and health-insurance regulations as well as  cultural differences [18]. Both issues, the literature inconsistencies due to including many treatment types collectively under the term ‘CAM’, and the large country-specific differences, call for the study of usage patterns of alternative medicine per country and per treatment on the basis of a clear, well-defined terminology.

Within this article, the focus lies on studying the factors related to the use of a specific subcategory of CAM, herbal medicine (HM), in Germany. HM was often found to be among the most popular and strongest growing forms of CAM [1–5, 10–12]. To name just one example, in 2007 Gardiner et al. reported a prevalence of 19% of respondents (US adult population) having used HM in the past 12 months [19]. However, as with CAM, the prevalence rates in EU countries vary significantly (6% - 48%), due to similar reasons, i.e. the unclear terminology and definition of HM used in different studies and country-specific variations [12, 16]. Indeed, previous work often considered HM combined with other treatments (e.g. as part of CAM [8, 13, 20, 21], together with further ‘natural health products’, or dietary supplements, such as mega-vitamins, etc. [22–26]. Moreover, previous studies solely analysing HM often focused on a specific part of the population (e.g. cancer patients, surgical patients, pregnant women, the elderly, etc. [27–32]). However, it is very difficult to generalise use-related factors and reasons related to, e.g. cancer patients, to the general population. Werneke et al. [33], for example asked cancer patients for their reasons for taking HM or dietary supplements and were told to ‘fight cancer’ or ‘boost immune system’. These are, of course, relevant reasons for this specific target group but may also provide a limited insight for the general population. This is an important issue, because the aims for HM use were shown to go beyond the mere treatment of an illness, and included preventing it and maintaining/promoting health [34, 35], i.e. many users who currently do not even have a specific condition still use HM.

In our work, we examine the factors and reasons relevant for the use of HM, applying an explorative focus group (FG) study in Germany. Furthermore, the aims of and reasons for preferred HM use, the role of the type of illness, and sources of information for different age groups (see also results on HM prevalence rates in refs. [19, 22, 36, 37]) are explored. In view of the terminology issues mentioned above, HM is defined as all plant-derived products including their natural form, as well as pills derived from extracts. The FG approach is ideal to explore complex human behaviour, attitudes, and motivation [38–42]. In using it, the aim is not to address a statistically-representative pool of opinions. Rather, our goal is to generate a set of deep data and peoples’ insights from the group discussions. We therefore argue that our results can complement more quantitative results on reasons and factors relevant for the use of HM.

## Methods

### Research methodology and data collection method

To examine the factors and reasons associated with the use of HM, we followed a qualitative descriptive method [43], and focus groups were used for data collection. The optimum number of focus groups and participants per group is not strictly defined a priori and debated in the literature [40, 44–46]. In our study, we had a total of six focus groups with six to nine members each, and followed the recommendations given by Krüger & Casey [46].

### Recruitment and participants

To recruit the participants of the focus groups, articles in local and regional newspapers were published in July 2016. Furthermore, flyers were distributed at different places of interest, including the local hospital, the authors’ scientific institute, and specific online communities. The target group of our focus groups was the general population, but specifically people with a general interest in and/or experiences with HM that were 18 years or older and German-speaking. The selection of participants in line with these criteria provided focus groups of participants who were aligned, as regards a general interest in the use of HM, to our core questions. At the same time, the focus groups chosen this way offered a bandwidth of different user experiences. On the basis of their age, the recruited participants (46 in total) were allocated to an age-specific focus group discussion. Thus, we conducted two focus group discussions each for people who were 18–35 years (defined as ‘young’), 36–59 years (‘middle-aged’), and > 60 years (‘elderly’).

### Focus group procedure

All the participants were informed about the content and purpose of the study prior to the FG and joined voluntarily, i.e. they gave informed consent. Our six focus group discussions were held at the authors’ scientific institute between 5^th^ July 2016 and 22^nd^ July 2016. Each focus group discussion lasted approximately two hours and was moderated by two of the authors (ANW and KM) using a semi-structured guideline. Both moderators were experienced in moderating group discussions. Furthermore, a research assistant joined each session and operated a digital voice recorder and also took notes on facial expressions/behaviour that could not be audio-taped. Each focus group discussion began with an introduction round, in which the participants, the moderators, as well as the research assistant, introduced themselves. The moderators guided the discussion following a questioning route, encouraging the participants to speak freely and to openly share their views [46, 47]. They also answered questions regarding the definition of HM and, with this, a common understanding of HM for all participants of the FG discussions was established. The key topics of our questioning route considered personal experiences in the use of HM, the reasons for using HM, and sources of information about the use. At the end of each focus group discussion, each participant provided socio-demographic and health-related data in a questionnaire. Each participant was given €25 as an incentive.

### Data analysis

The recordings of the six discussions were transcribed verbatim. After cross-checking the transcripts against the records by the first author twice, and several readings of the transcripts and memos, the transcripts were analysed using the ‘MaxQDA’ [48] qualitative data analysis software. It offered the option to structure, systemise, and compare the contents of transcripts from focus group discussions [49]. To analyse our data, qualitative content analysis was used [50], following the deductive-inductive technique of coding the data and building categories to describe and explain it (for further details of this approach, the reader is referred to ref. [51]). In an initial step, the coding system, with relevant reasons and attitudes concerning the use of HM, was developed by ANW, based on a literature review (deductive). In a second step, the coding system was refined inductively based on relevant text passages, relating to the key questions of our study. Specifically, we adapted the coding system by analysing the transcripts of three of the focus group discussions, repeating this procedure until no more changes in the coding system were noted. Then, we adapted the coding system further by analysing the transcripts of all the discussions, repeating this again until no more changes were noted. The entire coding process was accompanied by discussions off all authors, and the completed coding system was reviewed separately by a second member of our team (AEK).

### Trustworthiness

Following the guidelines suggested by Lincoln and Guba [52], in this study the following techniques were employed to maintain the trustworthiness of our findings. Well-established data collection and analysis methods were used to enhance *credibility*. During the entire research process, more than one researcher was involved, as described in detail above. All involved researchers were experienced in the moderation of group discussions. In particular, the FG discussions were all audio taped and also protocolled. Moreover, the FG participants joined the discussions voluntarily, and therefore the basis for the participants to be honest and open was established. *Dependability* of our research was ensured by using a consistent approach for the data collection and methodology, as described in detail above. To maintain *confirmability* and reduce the influence of subjective bias, during the entire data collection and analysis period the researchers held frequent meetings, reflexive and critical discussions, and debriefings. This ensured that every step during the data analysis procedure was well-documented. Also, explicit quotations of different participants of the FGs are cited below to enhance confirmability of the findings of this study. For providing *transferability*, the research procedure, including data collection and analysis, was described as detailed as possible to enhance the transparency of the research design used. Additionally, important contextual information such as the period of time of the data collection sessions, their number and length, and the restrictions which have been used to recruit people was provided. Finally, the questioning route of the FG discussions can be found in the Additional file 1.

## Results

In this section, first details of the participants of the FGs are briefly reported; secondly, results of the key themes of the questioning route are reported, namely a description of the area of application of HM, reasons for its use, as well as information sources. The quotations which will be subsequently presented were carefully selected to be representative for the topic. Note that certain passages in the quotations had to be anonymised. For each quote, we specify the focus group no. according to Table 1.

**Table 1:** Composition of the six focus groups

Group No.	Age Group	Age Range (Years)	No. of Participants (Female/Male)
1	Young	18–28	6 (2/4)
2	Young	18–28	7 (4/3)
3	Middle-aged	41–59	8 (5/3)
4	Middle-aged	49–59	9 (9/0)
5	Elderly	62–88	8 (3/5)
6	Elderly	66–82	8 (5/3)
Total		18–88	46 (28/18)

### Participants

A total of 46 people responded to our above-described announcement and participated in the six focus groups. These participants have an average age of 51.8 years and were predominantly women, i.e. 60.8%, see below for discussion. Table 1 provides an overview of the focus groups, including their classification according to age including the age range of the participants, the number of participants and gender distribution per group. Additionally, the mean values of the age of the participants per age group are noted: 24.0 (standard deviation, SD = 3.9), 53.6 (SD = 4.8), and 72.3 (SD = 8.1) for young, middle-aged, and elderly, respectively.

### Aims of use

In the FG discussions, three major aims of the use of HM (see Table 2) were revealed, namely to promote health, to prevent chronic or acute illness, and to treat them, which was the most important aim. There were specific differences by age group: Promoting health with HM was solely important for elderly participants, who mentioned this aspect four times, but it was not mentioned in the other groups. Prevention of chronic or acute illness with HM was especially important for middle-aged and elderly participants, but not for younger ones. For the latter, with the exception of a threat of a serious disease, preventative medication was clearly not relevant:

*I try to eat healthily, but I do not take herbal medicine as a preventative care, for not becoming ill later.* (FG No. 1).

**Table 2:** Overview of the different aims for the use of HM for all focus groups. The numbers represent the absolute frequency of mentioning a specific aim with multiple answers being possible

Aims	Young (*n* = 13)	Middle-Aged (*n* = 17)	Elderly (*n* = 16)	Total
Promote health	–	–	4	4
Prevent chronic or acute illness	1	4	8	13
Treat chronic or acute illness	12	18	19	49

Important for all age groups, and the most common aim for using HM, was treating an illness. This applied to treatments of both acute and chronic illnesses (Table 2).

### Role of type of illness

Another important aspect that influences participants´ choice of treatment with HM was the specific disease per se. In this context, participants mentioned a variety of illnesses for which they use HM as a preferred treatment method – these are summarised in Table 3.

**Table 3:** Absolute frequencies of indications mentioned for using HM (multiple answers were possible)

Indication	N
Head and chest cold	13
Flu infection	7
Sleeping disturbances and restlessness	6
Musculoskeletal issues	6
Gastrointestinal problems	4
Depression	3
Insect bites/itching	3
Allergies	3
High Cholesterol	2
Gynaecological/urological problems	2
Dermatitis	1
Immune system	1
Mental function	1
High blood pressure	1
Tinnitus	1
Blood glucose	1

As shown in Table 3, head and chest colds, flu infection, sleeping disturbances and musculoskeletal issues are the most frequently mentioned illnesses treated with HM. Notably, several participants also mentioned giving HM to their children. Furthermore, HM is seen as a starting treatment before resorting to treatment with conventional medicine (CM):

*In my family of six everyone is ill from time to time and, for this I always use plant medicine as a first treatment.* (FG No. 3)

Summarising the aspects in the discussions related to the limits of HM and border to CM, the participants mentioned serious diseases (diseases such as cancer, asthma, attention deficit hyperactivity disorder), receiving treatment during and after operations, severe pain, and a fast recovery as important factors:

*One of my sons is also ill (attention deficit hyperactivity disorder – Ed. note) and I have tried to use globules, plant remedies and many other things, because I refused synthetic drugs – this showed me that without synthetic drugs it actually does not work. However, if he has a cold or gastro-intestinal disease, he still gets something from my plant medicine chest. However, in the one specific case (of the chronic disease – Ed. note) it just did not work with anything that was tried, and then there is no other way. That is how it is, but it also convinced me of the synthetic route compared to plant medicine. It clearly showed me – stop, there is a limit. For this specific case, there is no way out.* (FG No. 3)

*So, I have two severe chronic illnesses, and for these two I believe there is no herb. For these you have to use real (medicine – Ed. note), whether one likes it or not. I already use teas, (…) for example to treat a bladder infection. (…) However, for real (heavy – Ed. note) diseases, I do not believe this to be so efficient.* (FG No. 5)

### Reasons for preferred use

Dissatisfaction with CM, and looking for alternative treatment methods as a consequence, was the most commonly-mentioned reason for using HM among the FG participants. Several participants provided detailed accounts of long-term illness histories, including failed conventional treatment efforts, frustration, and disappointments. Too many side-effects of CM, a lack of treatment effect, as well as dissatisfaction with the conventional doctor were issues mentioned in this context:

*I have suffered from neurodermatitis for several decades . Yes, the dermatologists prescribed cortisone for applying it to the skin. Cortisone, this makes the skin thinner, and the dermatologists, they provided me with a very bad prognosis, namely my skin will get thinner and the neurodermatitis will get worse and worse, and they felt sorry for me. During the holidays, one of my relatives gave me a book about folk medicine and there I read it, namely, that there is a plant for the skin, sarsaparilla was the name. I bought a special ointment in the pharmacy. I paid for it myself and used it for a while. It did me good. (..) and, for a long time, I have not had any problems. For decades now, I have not needed a dermatologist. This is, I believe, due to plant remedies.* (FG No. 3)

*(…) and I realised that, after taking all those pills, I had very strong side-effects, and that these were even worse than the symptoms I had before.* (FG No. 6)

The second most important reason for the use of HM provided in the FG discussions was a positive experience with using HM in the past, including treatment successes and a positive impact on health. This lead people to access HM again when needed, and to maintain this specific treatment approach:

*In the past, I have always suffered from a cold. I tried a lot, nothing helped, but then I read about plant saps. When using a specific plant sap, for maybe two weeks or a month, then one can prevent such issues. I did it, and lo and behold, all my cold symptoms, which I had regularly four or five times each winter, with a heavy flu, suddenly became less pronounced! I only had these once a year instead. The next winter, I used preventative care again and passed the whole winter without having a single cold. It really helped fabulously and since then I am convinced. I have now used plant sap for six years whenever necessary and I can regulate my entire physical health, like blood-sugar and cholesterol or similar things. When a doctor checks my blood, then I immediately realise that it works.* (FG No. 6)

Beyond the above-mentioned dissatisfaction with CM and positive experiences with HM in the past, in discussing reasons for the use of HM, participants mentioned several positive (‘healthy’) aspects and beliefs they associated with using HM: participants evaluated HM as being healthier, more natural, providing a higher tolerability, having few or no side-effects, showing an easier absorption of the ingredients by the body, and a better degradation. In addition to all these aspects, an apparent knowledge of the detailed contents of the HM played an important role for many participants. Being familiar with a plant, either because of knowing the name, or even because of cultivating the plant in the own garden, was said to provide a basis of trust for the users of HM. In contrast, when using chemically-synthesised drugs, participants discussed the issue that not knowing the contents lead them to distrust the treatment:

*Between a drug that is made from a plant, or one which is synthetic, I would always decide for the herbal one, simply because one always knows where one stands. If one reads the package of different medicines, I know only very few of the ingredients. (..) I personally would still always prefer to try this (HM) first before taking any other remedy which I do not exactly know what it is. I would just trust more that it could help. Maybe it is also a placebo effect, but I prefer to take this into account rather than putting something into my body when I really do not know what it is and that it is chemically-made.* (FG No. 2)

The FG discussion also clearly demonstrated the important influence of tradition and family history on the motivation for using HM: long-standing traditions in families and knowledge regarding “what helps” were discussed as being handed-down generation by generation. After dissatisfaction with CM, positive experiences with HM and the positive aspects/beliefs, the traditional feature, i.e. “it has always been done this way” was the fourth most important reason for preferring HM as a treatment method:

*Well, I mean this actually started when we were still kids. If you were ill, then you just got your elderflower juice or lime-blossom tea and I mean they also just have an effect; this is something you take along with you when you get older, and for your own small children, before one always gives them strong medicine, one also tries the things learned from one’s own parents.* (FG No. 3)

*As a child, I learned from my grandmother what she learned from her grandmother. It started with the fact that even as a child, I also drank lime-blossom tea and mint tea. My grandmother always said that these are for the stomach and cough. During summer, the cold lime-blossom tea, then you will not get a cough and, during winter, a hot one, then the flu disappears. From this, I started to investigate further and now I just take everything (herbal – Eds. Note).* (FG No. 4)

### Sources of information

The aforementioned influence of family traditions on the use of HM can also be examined from a different viewpoint, namely when considering the sources of information about HM summarised in Table 4. Thereby, medicinal experts were mentioned only seven times over all the FGs and have about the same importance as information originating from within the family. In addition to this, a significant source of knowledge for users is provided by independent reading. Note that the potential side-effects and/or harm due to HM use were not mentioned.

**Table 4:** Sources of Information. The numbers represent the absolute frequency of mentioning a specific information source

Sources	N
Book/magazine	10
Parents/grandparents	7
Medicinal expert	7
Friends	3
Education/information event	3
Trial and error	2

## Discussion

On the basis of a focus group methodology, our study explored the factors and reasons for consumers using HM in the general population. The first important finding of our study is that the FG participants were predominantly (60.8%) female. When recruiting the FG participants, one important inclusion criterion was a general interest in and/or experiences with HM. The fact that this criterion predominantly attracted female participants is seen as an indication that the user of HM is primarily female, similar to previous results [19, 36, 37, 53, 54].

Regarding the aims when using HM that were discussed in the FGs, participants explained that treating an illness was the most common aim for using HM. Preventing an illness and promoting health were less important. It is possible that the latter two aspects are covered more by the use of dietary supplements or other forms of CAM therapies [22, 25] and not by HM. It may also reflect the idea that health-orientated behaviour (such as drug use for preventing illness or promoting health) becomes relevant for people only when they are confronted with a substantial health-threat, and not as long as they are healthy. Indeed, participants of the young, middle-aged and elderly FGs discussed different aims for the use of HM, which indicates that health awareness and maintenance become increasingly important with people’s growing age. These were the only differences found in the discussions of the studied age groups.

With respect to most common diseases for which participants discussed the use of HM, we can confirm literature results. Gardiner et al. [19], and Kennedy [23] arrived at similar conclusions in their studies, i.e. that head and chest colds are the most common diseases for the use of HM. We also note similarities between previous findings on CAM usage and results from our FG discussions on HM, such as the use of HM/CAM as a treatment before resorting to CM [55].

As regards the reasons why HM is preferred as a treatment method, in our FGs the most commonly-mentioned ones were dissatisfaction with CM, positive experiences with HM in the past, and positive aspects and beliefs associated with HM. As discussed in the literature on CAM, the first reason can be categorised as a ‘push’ factor (negative aspects/beliefs regarding CM), and the latter two as so-called ‘pull’ factors (positive aspects/beliefs regarding HM) [56]. In line with previous findings [21, 56], in our FG discussions, ‘push’ factors were associated more closely with initially using HM, whereas ‘pull’ factors were discussed more for the motivation to maintain the use of HM behaviour. Thus, different reasons vary in their degree of contributing to the initial and maintained use of HM.

Another specific aspect was the importance of family traditions in the discussions about HM. This became clear in the discussions about the reasons for the use of HM (“it was always done this way”) and family members are also one of the most important sources of information concerning HM.

In our FG discussions, HM often appeared as a form of self-medicated treatment for which many users aim to become experts themselves by, e.g. consulting books or family members. Such sources of information are often considered to be even more important than recommendations by a doctor/practitioner or other experts – this is in line with previous results concerning information sources of HM [28, 36, 57, 58], CAM [7, 59] and on natural health products [22]. Note that Bardia et al. found that only one-third of the study participants used a majority of HM treatments in accordance with evidence-based indications [37]. In this context, it is also relevant that FG participants hardly discussed the possible side-effects of HM or negative interactions with other drugs. Quite on the contrary, HM was discussed as being harmless and having many advantages in the FGs. Many herbal products have evidence-based good efficiencies and safety profiles [60, 61], but one must be aware of various adverse effects, such as toxicity, over-dosage, herb contamination, and especially herb-herb or herb-drug interactions [62–66]. Studies have shown an apparent lack of risk awareness of HM users, as the majority (82%) believes that there are no interactions of HM with other types of medicine [36].

Even though the FG methodology has various positive aspects, such as ensuring a clear terminology and mutual understanding of what constitutes HM for all participants of the study, we also note the limitations of our approach. First of all, results from FG studies are difficult to quantify [39], although a high standard of research (see methods section) was followed to achieve reliability and validity, the qualitative analysis of the FG transcripts may still be influenced by the authors. Furthermore, results from descriptive qualitative research cannot be generalized easily, as the aim is to provide deep data and insight from the discussions rather than addressing a statistically relevant data pool. Moreover, we expect that our results are not easily transferable to other countries because of the vast differences in healthcare systems, regulations, and cultural beliefs. It is also noted that our study does not consider the general population in Germany because all of the FGs were conducted in the same regional area and participants were selected based on a general interest in HM.

## Conclusion

We conclude that HM was found to be used predominately for treating mild to moderate diseases (all age groups) and to prevent illnesses/promote health (only elderly participants), and that participants were aware of its limits. Nevertheless, the combination of self-medication, non-expert consultation and missing risk awareness reported here is potentially harmful, especially if people do not report the HM use to their doctor, which is a phenomenon frequently discussed in literature [8, 19, 23, 36, 58, 67, 68]. This issue is problematic, especially for elderly users, who appeared to be more aware of health-related issues, but also use more prescribed and non-prescribed medicine compared to younger ones [67–70]. It is therefore necessary that government bodies, doctors, and pharmaceutical companies aim to establish a certain minimum level of consumer awareness regarding the side and interaction effects of HM. It is equally important that these health-related decision-makers are aware of the dissatisfaction with CM, this being the most important reason for a preferred use of HM. Looking ahead, a consistent terminology and common set of CAM definitions, for example, what exactly constitutes HM as a form of treatment and whether, or to what extent, it is part of CAM, would be an important step towards more validity and comparability in this field [32]. Building on this, further well-designed research is necessary to obtain a detailed picture of prevalence rates, use-related factors, and reasons for the usage of HM.

## Additional file

Additional file 1: Questioning route. Description of data: The questioning route used in the focus group discussions is shown. (DOCX 13 kb)

## Abbreviations

CAM: Complementary and alternative medicine
CM: Conventional medicine
FG: Focus group
HM: Herbal medicine
