# Supplementary material for: Why people use herbal medicine: insights from a focus-group study in Germany
Source: BMC Complement Altern Med. 2018 Mar 15;18:92. doi: 10.1186/s12906-018-2160-6 (PMC5855977; doi:10.1186/s12906-018-2160-6)
Supplement: Supplementary file 1 — Questioning route. Description of data: The questioning route used in the focus group discussions is shown. (DOCX 13 kb) [file 12906_2018_2160_MOESM1_ESM.docx]

**Supplemental Material to “Why People Use Herbal Medicine: Insights from a Focus-Group Study in Germany”**

**Alexandra N. Welz, Agnes Emberger-Klein, Klaus Menrad**

TUM Campus Straubing for Biotechnology and Sustainability, Weihenstephan-Triesdorf University of Applied Sciences, Petersgasse 18, 94315 Straubing, Germany

Email: alexandra.welz@hswt.de; agens.emberger-klein@hswt.de; klaus.menrad@hswt.de

Correspondence should be addressed to Klaus Menrad: klaus.menrad@hswt.de

**ADDITIONAL FILE 1**

Questioning route of the focus group discussions:

- What do you think about plant remedies? Do you have a specific viewpoint with respect to using them?
- Which personal experiences do you have with the use of herbal medicine in your lifetime?
- Why are you using plant remedies?
  - Due to specific illnesses? What is the impact of the illnesses, especially comparing mild vs. strong ones? Are you using them in a preventive way or for an acute illness?
  - Are you using them in specific situations?
  - Did you have a specific reason when initiating the use of plant medicine?
  - Do you have other reasons?
- How do you inform yourself about the effect of plant medicine?
